# Crystal structure of 2-(4-chloro­benzamido)­benzoic acid

**DOI:** 10.1107/S2056989015017879

**Published:** 2015-10-17

**Authors:** Rodolfo Moreno-Fuquen, Vanessa Melo, Javier Ellena

**Affiliations:** a Departamento de Química - Facultad de Ciencias Naturales y Exactas, Universidad del Valle, Apartado 25360, Santiago de Cali, Colombia; b Instituto de Física de São Carlos, IFSC, Universidade de São Paulo, USP, São Carlos, SP, Brazil

**Keywords:** crystal structure, carb­oxy­lic acid, amide, hydrogen bonding

## Abstract

In the title mol­ecule, C_14_H_10_ClNO_3_, the amide C=O bond is *anti* to the *o*-carb­oxy substituent in the adjacent benzene ring, a conformation that facilitates the formation of an intra­molecular amide-N—H⋯O(carbon­yl) hydrogen bond that closes an *S*(6) loop. The central amide segment is twisted away from the carb­oxy- and chloro-substituted benzene rings by 13.93 (17) and 15.26 (15)°, respectively. The most prominent supra­molecular inter­actions in the crystal packing are carb­oxy­lic acid-H⋯O(carbox­yl) hydrogen bonds that lead to centrosymmetric dimeric aggregates connected by eight-membered {⋯HOC=O}_2_ synthons.

## Related literature   

For our studies on the effects of substituents on the structures of *N*-(ar­yl)-amides, see: Moreno-Fuquen *et al.* (2014 ▸, 2015 ▸). For benzanilide properties, see: Nuta *et al.* (2013 ▸); Leander (1992 ▸); Ahles *et al.* (2004 ▸). For related structures, see: Saeed *et al.* (2008 ▸, 2010 ▸); Rodrigues *et al.* (2011 ▸). For hydrogen bonding, see: Desiraju & Steiner (1999 ▸), Nardelli (1995 ▸).
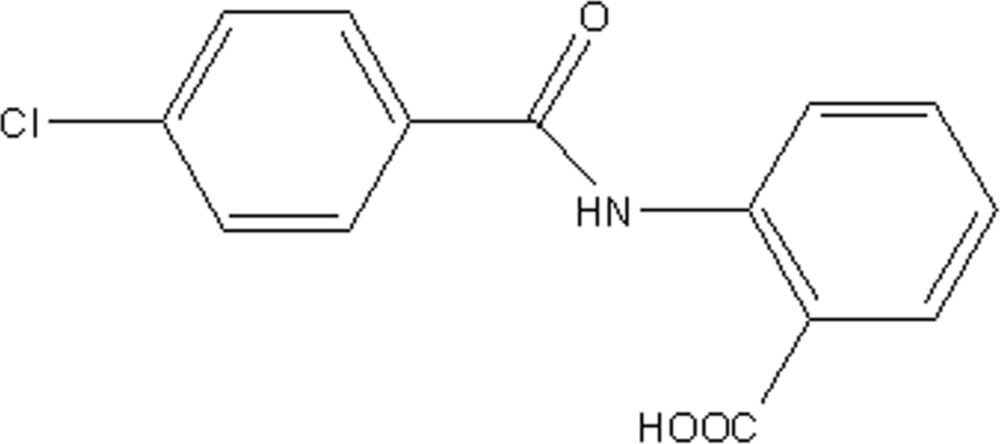


## Experimental   

### Crystal data  

C_14_H_10_ClNO_3_
*M* *_r_* = 275.69
Monoclinic 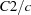

*a* = 26.8843 (10) Å
*b* = 5.0367 (2) Å
*c* = 20.9264 (12) Å
β = 117.489 (2)°
*V* = 2513.7 (2) Å^3^
*Z* = 8
Mo *K*α radiation
μ = 0.31 mm^−1^
*T* = 295 K
0.40 × 0.08 × 0.06 mm

### Data collection  

Nonius KappaCCD diffractometer
4248 measured reflections
2295 independent reflections
1049 reflections with *I* > 2σ(*I*)
*R*_int_ = 0.057

### Refinement  

*R*[*F* ^2^ > 2σ(*F* ^2^)] = 0.043
*wR*(*F* ^2^) = 0.132
*S* = 0.92
2295 reflections
176 parameters
H atoms treated by a mixture of independent and constrained refinement
Δρ_max_ = 0.20 e Å^−3^
Δρ_min_ = −0.19 e Å^−3^

### 

Data collection: *COLLECT* (Nonius, 2000 ▸); cell refinement: *HKL* *SCALEPACK* (Otwinowski & Minor, 1997 ▸); data reduction: *HKL* *DENZO* (Otwinowski & Minor, 1997 ▸) and *SCALEPACK*; program(s) used to solve structure: *SHELXS97* (Sheldrick, 2008 ▸); program(s) used to refine structure: *SHELXL2014*/7 (Sheldrick, 2015 ▸); molecular graphics: *ORTEP-3 for Windows* (Farrugia, 2012 ▸) and *Mercury* (Macrae *et al.*, 2006 ▸); software used to prepare material for publication: *SHELXL2014*/7.

## Supplementary Material

Crystal structure: contains datablock(s) I, global. DOI: 10.1107/S2056989015017879/tk5391sup1.cif

Structure factors: contains datablock(s) I. DOI: 10.1107/S2056989015017879/tk5391Isup2.hkl

Supporting information file. DOI: 10.1107/S2056989015017879/tk5391Isup3.cml

**Fig. 1.**
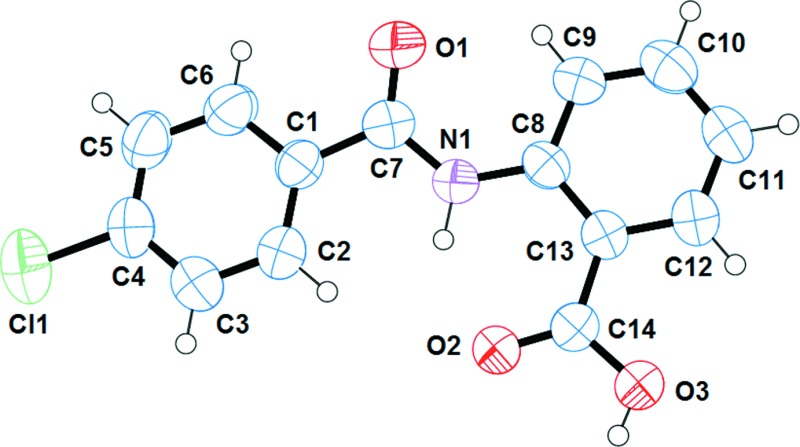
The molecular structure of (I) with displacement ellipsoids drawn at the 50% probability level. H atoms are shown as spheres of arbitrary radius.

. DOI: 10.1107/S2056989015017879/tk5391fig1.tif
The mol­ecular structure of (I) with displacement ellipsoids drawn at the 50% probability level. H atoms are shown as spheres of arbitrary radius.

CCDC reference: 1427117

Additional supporting information:  crystallographic information; 3D view; checkCIF report

## Figures and Tables

**Table 1:** Hydrogen-bond geometry (, )

*D*H*A*	*D*H	H*A*	*D* *A*	*D*H*A*
N1NH1O2	0.93(3)	1.96(3)	2.678(3)	133(3)
O3OH3O2^i^	0.82	1.83	2.645(3)	175

Symmetry code: (i) 
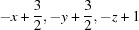
.

## supplementary crystallographic information

### S1. Comment

The crystal structure determination of 2-(4-chlorobenzamido)benzoic acid, (I), was investigated in a continuation of our studies on substituted *N*-phenyl benzamides which have been synthesized from picryl esters. This study was also performed to study the effect of substituents on the structures of benzanilides (Moreno-Fuquen *et al.*, 2014, 2015). Benzanilide systems are used as antimicrobial drugs (Nuta *et al.*, 2013), as anticonvulsants (Leander, 1992) or as treatment for patients with prostate carcinoma (Ahles *et al.*, 2004). Structures of similar molecules were compared with (I), *i.e*. 4-chloro-*N*-(2-methoxyphenyl)benzamide (Saeed *et al.*, 2010), 4-chloro-*N*-phenylbenzamide (Rodrigues *et al.*, 2011) and 4-chloro-*N*-(*o*-tolyl)benzamide (Saeed *et al.*, 2008). The molecular structure of (I) is shown in Fig. 1. The C═O bond is *anti* to the *o*-carboxy substituent in the benzoyl ring. The N—H and C=O bonds in the central amide group are also *anti* to each other. Comparing (I) with the three aforementioned structures reveals that significant differences in bond lengths and bond angles are not observed. The central amide segment (C1-C7(O1)-N1-C8) is twisted away from the carboxy- and chloro-substituted benzene rings by 13.93 (17) and 15.26 (15)°, respectively. Molecules of (I) are held together by intermolecular O—H···O hydrogen bonds of moderate strength (Desiraju & Steiner, 1999). The O3 atom is linked to O2^i^ atom (*i* = -*x*+3/2, -*y*+3/2, -*z*+1) with O···O distances of 2.645 (2), (see Table 1, Nardelli, 1995). Except for the presence of hydrogen bonding in the formation of dimer, no other significant intermolecular interactions are observed in the structure.

### S2. Experimental

2,4,6-Trinitrophenyl 4-chlorobenzoate (0.060 g, 0.163 mmol) and 2-carboxyaniline (0.045 g, 0.328 mmol) were dissolved in toluene (15 ml) and stirred for 6 h under reflux. On completion of the reaction part of the solvent was evaporated and a crystalline yellow solid was obtained; m.p. 470 (1) K.

### S3. Refinement

All H-atoms were located in difference Fourier maps and were positioned geometrically [C—H = 0.93 Å] and were refined using a riding-model approximation with *U*_iso_(H) constrained to 1.2*U*_eq_(C). The O-bound H atoms was similarly fixed with O—H = 0.82 Å, and with *U*_iso_(H) constrained to 1.5*U*_eq_(O). The N-bound H atom was found from the Fourier maps and was refined freely.

### Crystal data

**Table:**

C_14_H_10_ClNO_3_	*D*_x_ = 1.457 Mg m^−^^3^
*M**_r_* = 275.69	Melting point: 470(1) K
Monoclinic, *C*2/*c*	Mo *K*α radiation, λ = 0.71073 Å
*a* = 26.8843 (10) Å	Cell parameters from 2553 reflections
*b* = 5.0367 (2) Å	θ = 3.1–25.4°
*c* = 20.9264 (12) Å	µ = 0.31 mm^−^^1^
β = 117.489 (2)°	*T* = 295 K
*V* = 2513.7 (2) Å^3^	Needle, yellow
*Z* = 8	0.40 × 0.08 × 0.06 mm
*F*(000) = 1136	

### Data collection

**Table:**

Nonius KappaCCD diffractometer	1049 reflections with *I* > 2σ(*I*)
Radiation source: fine-focus sealed tube	*R*_int_ = 0.057
Graphite monochromator	θ_max_ = 25.4°, θ_min_ = 3.1°
CCD rotation images, thick slices scans	*h* = −31→32
4248 measured reflections	*k* = −6→6
2295 independent reflections	*l* = −25→24

### Refinement

**Table:**

Refinement on *F*^2^	Primary atom site location: structure-invariant direct methods
Least-squares matrix: full	Secondary atom site location: difference Fourier map
*R*[*F*^2^ > 2σ(*F*^2^)] = 0.043	Hydrogen site location: inferred from neighbouring sites
*wR*(*F*^2^) = 0.132	H atoms treated by a mixture of independent and constrained refinement
*S* = 0.92	*w* = 1/[σ^2^(*F*_o_^2^) + (0.0632*P*)^2^] where *P* = (*F*_o_^2^ + 2*F*_c_^2^)/3
2295 reflections	(Δ/σ)_max_ < 0.001
176 parameters	Δρ_max_ = 0.20 e Å^−^^3^
0 restraints	Δρ_min_ = −0.19 e Å^−^^3^

### Special details

**Table:**

Geometry. All esds (except the esd in the dihedral angle between two l.s. planes) are estimated using the full covariance matrix. The cell esds are taken into account individually in the estimation of esds in distances, angles and torsion angles; correlations between esds in cell parameters are only used when they are defined by crystal symmetry. An approximate (isotropic) treatment of cell esds is used for estimating esds involving l.s. planes.
Refinement. Refinement of *F*^2^ against ALL reflections. The weighted *R*-factor *wR* and goodness of fit *S* are based on *F*^2^, conventional *R*-factors *R* are based on *F*, with *F* set to zero for negative *F*^2^. The threshold expression of *F*^2^ > σ(*F*^2^) is used only for calculating *R*-factors(gt) *etc*. and is not relevant to the choice of reflections for refinement. *R*-factors based on *F*^2^ are statistically about twice as large as those based on *F*, and *R*- factors based on ALL data will be even larger.

### Fractional atomic coordinates and isotropic or equivalent isotropic displacement parameters (Å^2^)

**Table:**

	*x*	*y*	*z*	*U*_iso_*/*U*_eq_	
O1	0.74640 (9)	−0.2731 (4)	0.30942 (12)	0.0824 (7)	
C5	0.90834 (14)	−0.0840 (7)	0.34839 (18)	0.0786 (9)	
H5	0.9265	−0.1847	0.3282	0.094*	
C10	0.58970 (13)	−0.0955 (6)	0.29315 (17)	0.0739 (9)	
H10	0.5647	−0.2205	0.2623	0.089*	
C9	0.64347 (12)	−0.0846 (5)	0.30083 (15)	0.0623 (8)	
H9	0.6543	−0.2008	0.2750	0.075*	
C2	0.85453 (13)	0.2133 (6)	0.40771 (18)	0.0766 (9)	
H2	0.8366	0.3155	0.4280	0.092*	
C11	0.57224 (13)	0.0750 (6)	0.33029 (18)	0.0754 (9)	
H11	0.5357	0.0667	0.3242	0.090*	
C6	0.85319 (14)	−0.1349 (6)	0.33124 (17)	0.0730 (9)	
H6	0.8344	−0.2716	0.2993	0.088*	
C3	0.90974 (14)	0.2649 (6)	0.42543 (18)	0.0801 (10)	
H3	0.9289	0.4004	0.4577	0.096*	
C4	0.93606 (13)	0.1173 (7)	0.39566 (17)	0.0701 (9)	
NH1	0.7536 (13)	0.280 (6)	0.3758 (17)	0.102 (11)*	
Cl1	1.00534 (4)	0.1835 (2)	0.41722 (6)	0.1062 (4)	
N1	0.73632 (10)	0.1205 (5)	0.35559 (12)	0.0592 (7)	
O3	0.67865 (8)	0.6206 (3)	0.46790 (10)	0.0688 (6)	
OH3	0.7015	0.7286	0.4949	0.103*	
O2	0.75129 (8)	0.5079 (3)	0.45041 (10)	0.0641 (6)	
C14	0.70192 (12)	0.4755 (5)	0.43671 (14)	0.0548 (7)	
C8	0.68169 (11)	0.1015 (5)	0.34751 (14)	0.0529 (7)	
C7	0.76554 (12)	−0.0609 (6)	0.33845 (15)	0.0599 (7)	
C13	0.66432 (11)	0.2748 (5)	0.38605 (14)	0.0535 (7)	
C1	0.82537 (12)	0.0120 (5)	0.36030 (15)	0.0568 (7)	
C12	0.60946 (12)	0.2573 (6)	0.37640 (15)	0.0664 (8)	
H12	0.5979	0.3718	0.4018	0.080*	

### Atomic displacement parameters (Å^2^)

**Table:**

	*U*^11^	*U*^22^	*U*^33^	*U*^12^	*U*^13^	*U*^23^
O1	0.0839 (16)	0.0631 (13)	0.1073 (17)	−0.0111 (11)	0.0502 (14)	−0.0225 (12)
C5	0.075 (2)	0.090 (2)	0.083 (2)	0.0100 (19)	0.047 (2)	−0.004 (2)
C10	0.063 (2)	0.071 (2)	0.072 (2)	−0.0093 (16)	0.0174 (18)	0.0023 (17)
C9	0.063 (2)	0.0595 (17)	0.0591 (19)	−0.0054 (15)	0.0242 (16)	−0.0031 (15)
C2	0.066 (2)	0.081 (2)	0.090 (2)	−0.0010 (17)	0.0417 (18)	−0.0156 (19)
C11	0.056 (2)	0.083 (2)	0.083 (2)	−0.0118 (18)	0.0279 (18)	−0.0009 (19)
C6	0.080 (2)	0.072 (2)	0.074 (2)	0.0010 (17)	0.0420 (19)	−0.0089 (16)
C3	0.066 (2)	0.085 (2)	0.089 (2)	−0.0102 (17)	0.035 (2)	−0.0121 (19)
C4	0.0581 (19)	0.083 (2)	0.073 (2)	0.0059 (17)	0.0343 (17)	0.0122 (18)
Cl1	0.0657 (6)	0.1390 (9)	0.1189 (8)	0.0021 (5)	0.0469 (6)	0.0093 (6)
N1	0.0576 (16)	0.0555 (15)	0.0681 (16)	−0.0068 (13)	0.0322 (13)	−0.0118 (13)
O3	0.0622 (13)	0.0726 (12)	0.0763 (13)	−0.0053 (10)	0.0360 (11)	−0.0183 (11)
O2	0.0560 (13)	0.0685 (12)	0.0694 (13)	−0.0070 (10)	0.0304 (11)	−0.0138 (10)
C14	0.0581 (19)	0.0553 (17)	0.0522 (18)	0.0026 (15)	0.0264 (16)	0.0028 (14)
C8	0.0513 (17)	0.0527 (15)	0.0506 (16)	−0.0041 (13)	0.0202 (14)	0.0055 (14)
C7	0.068 (2)	0.0570 (18)	0.0555 (18)	0.0000 (16)	0.0298 (16)	0.0024 (15)
C13	0.0515 (17)	0.0564 (16)	0.0514 (17)	−0.0007 (14)	0.0228 (14)	0.0040 (14)
C1	0.0608 (19)	0.0581 (17)	0.0554 (18)	0.0033 (15)	0.0302 (16)	0.0025 (14)
C12	0.0564 (19)	0.074 (2)	0.071 (2)	−0.0005 (16)	0.0314 (16)	0.0024 (16)

### Geometric parameters (Å, º)

**Table:**

O1—C7	1.219 (3)	C6—H6	0.9300
C5—C4	1.371 (4)	C3—C4	1.360 (4)
C5—C6	1.379 (4)	C3—H3	0.9300
C5—H5	0.9300	C4—Cl1	1.734 (3)
C10—C11	1.378 (4)	N1—C7	1.357 (3)
C10—C9	1.380 (4)	N1—C8	1.402 (3)
C10—H10	0.9300	N1—NH1	0.93 (3)
C9—C8	1.401 (4)	O3—C14	1.315 (3)
C9—H9	0.9300	O3—OH3	0.8200
C2—C3	1.378 (4)	O2—C14	1.233 (3)
C2—C1	1.382 (4)	C14—C13	1.476 (4)
C2—H2	0.9300	C8—C13	1.406 (4)
C11—C12	1.373 (4)	C7—C1	1.501 (4)
C11—H11	0.9300	C13—C12	1.396 (4)
C6—C1	1.377 (4)	C12—H12	0.9300
			
C4—C5—C6	119.1 (3)	C5—C4—Cl1	119.4 (3)
C4—C5—H5	120.5	C7—N1—C8	128.6 (3)
C6—C5—H5	120.5	C7—N1—NH1	118 (2)
C11—C10—C9	121.4 (3)	C8—N1—NH1	113 (2)
C11—C10—H10	119.3	C14—O3—OH3	109.5
C9—C10—H10	119.3	O2—C14—O3	121.3 (3)
C10—C9—C8	120.0 (3)	O2—C14—C13	124.4 (3)
C10—C9—H9	120.0	O3—C14—C13	114.3 (3)
C8—C9—H9	120.0	C9—C8—N1	121.3 (3)
C3—C2—C1	121.0 (3)	C9—C8—C13	119.0 (3)
C3—C2—H2	119.5	N1—C8—C13	119.7 (2)
C1—C2—H2	119.5	O1—C7—N1	124.0 (3)
C12—C11—C10	119.1 (3)	O1—C7—C1	120.8 (3)
C12—C11—H11	120.5	N1—C7—C1	115.1 (3)
C10—C11—H11	120.5	C12—C13—C8	119.1 (3)
C1—C6—C5	121.5 (3)	C12—C13—C14	118.3 (3)
C1—C6—H6	119.2	C8—C13—C14	122.6 (3)
C5—C6—H6	119.2	C6—C1—C2	117.9 (3)
C4—C3—C2	119.8 (3)	C6—C1—C7	117.3 (3)
C4—C3—H3	120.1	C2—C1—C7	124.9 (3)
C2—C3—H3	120.1	C11—C12—C13	121.5 (3)
C3—C4—C5	120.7 (3)	C11—C12—H12	119.2
C3—C4—Cl1	119.9 (3)	C13—C12—H12	119.2
			
C11—C10—C9—C8	0.4 (4)	N1—C8—C13—C14	−1.4 (4)
C9—C10—C11—C12	−0.7 (5)	O2—C14—C13—C12	179.1 (3)
C4—C5—C6—C1	0.2 (5)	O3—C14—C13—C12	−0.2 (3)
C1—C2—C3—C4	0.4 (5)	O2—C14—C13—C8	−0.8 (4)
C2—C3—C4—C5	−0.4 (5)	O3—C14—C13—C8	179.9 (2)
C2—C3—C4—Cl1	179.4 (2)	C5—C6—C1—C2	−0.2 (4)
C6—C5—C4—C3	0.1 (5)	C5—C6—C1—C7	−179.6 (3)
C6—C5—C4—Cl1	−179.7 (2)	C3—C2—C1—C6	−0.1 (4)
C10—C9—C8—N1	−178.9 (2)	C3—C2—C1—C7	179.3 (3)
C10—C9—C8—C13	0.1 (4)	O1—C7—C1—C6	15.5 (4)
C7—N1—C8—C9	−18.7 (4)	N1—C7—C1—C6	−166.6 (2)
C7—N1—C8—C13	162.3 (3)	O1—C7—C1—C2	−163.9 (3)
C8—N1—C7—O1	3.2 (5)	N1—C7—C1—C2	14.0 (4)
C8—N1—C7—C1	−174.6 (2)	C10—C11—C12—C13	0.4 (4)
C9—C8—C13—C12	−0.3 (4)	C8—C13—C12—C11	0.1 (4)
N1—C8—C13—C12	178.7 (2)	C14—C13—C12—C11	−179.8 (2)
C9—C8—C13—C14	179.6 (2)		

### Hydrogen-bond geometry (Å, º)

**Table:**

*D*—H···*A*	*D*—H	H···*A*	*D*···*A*	*D*—H···*A*
N1—NH1···O2	0.93 (3)	1.96 (3)	2.678 (3)	133 (3)
O3—OH3···O2^i^	0.82	1.83	2.645 (3)	175

Symmetry code: (i) −*x*+3/2, −*y*+3/2, −*z*+1.
