# Supplementary figures and images for: Crystal structure of 2-(4-chloro­benzamido)­benzoic acid
Source: Acta Crystallogr E Crystallogr Commun. 2015 Oct 17;71(Pt 11):o856–7. doi: 10.1107/S2056989015017879 (PMC4645063; doi:10.1107/S2056989015017879)

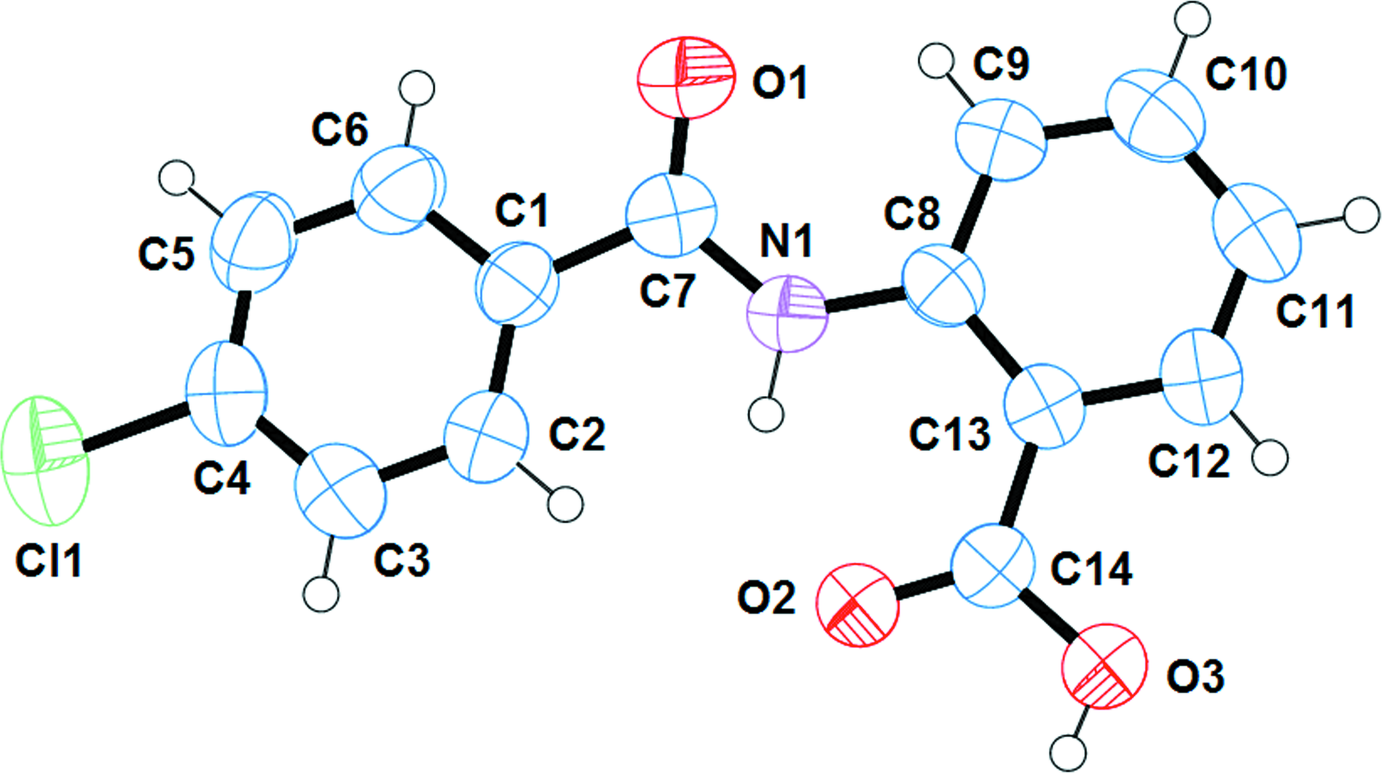

Supplement: Supplementary file 4 [file e-71-0o856-fig1.tif]
